# Economic impact of powered stapler in video-assisted thoracic surgery lobectomy for lung Cancer in a Chinese tertiary hospital: a cost-minimization analysis

**DOI:** 10.1186/s13561-022-00359-x

**Published:** 2022-02-09

**Authors:** Yang Cao, Fang Xiong, Xiaozhe Xia, Pengjuan Gu, Qinghong Wang, Aiping Wu, Huan Zhan, Wendong Chen, Zhaoxin Qian

**Affiliations:** 1 Xiangya Hospital, Central South University, Changsha, China; 2 Changsha Normin Health Technology Ltd, Changsha, China; 3 Normin Health Consulting Ltd, Toronto, Canada

**Keywords:** Powered stapler, VATS lobectomy, Lung cancer, Hospital costs, Cost-minimization analysis

## Abstract

**Background:**

To assess the economic impact of powered stapler use in video-assisted thoracic surgery (VATS) lobectomy for lung cancer in a Chinese tertiary care hospital.

**Methods:**

This study identified 388 patients who received VATS lobectomy using the ECHELON powered stapler (*n* = 296) or the ECHELON manual stapler (*n* = 92) for lung cancer in a Chinese tertiary hospital. Multiple generalized linear regression analyses were conducted using data on hospital costs and patient characteristics to develop predictive equations for hospital costs in a cost-minimization analysis (CMA) model comparing hospital costs associated with the ECHELON powered stapler and the ECHELON manual stapler. CMA model was used to conduct scenario analysis to compare the ECHELON powered stapler with another manual stapler (Victor Medical).

**Results:**

The multiple generalized linear regression analyses identified that using the ECHELON powered stapler in VATS lobectomy for lung cancer was associated with significantly lower drug costs than using the ECHELON manual stapler (coefficient − 0.256, 95% confidence interval: − 0.375 to − 0.139). The CMA model estimated that the ECHELON powered stapler could save hospital costs by ¥1653 when compared with the ECHELON manual stapler (¥65,531 vs. ¥67,184). The use of the ECHELON powered stapler also saved hospital costs by ¥4411 when compared with the Victor Medical manual stapler (¥65,531 vs. ¥69,942) in the scenario analysis.

**Conclusions:**

Compared to the two manual staplers used for VATS lobectomy for lung cancer in a Chinese tertiary hospital, the ECHELON powered stapler had 100% probability to save total hospital costs under present prices of the three staplers according to the CMA.

**Supplementary Information:**

The online version contains supplementary material available at 10.1186/s13561-022-00359-x.

## Introduction

With its genesis as a “mechanical stitching device” in 1908, the stapler evolved from the “Fischer-Hul̈tl stapler”, which weighed 5 kg and took 2 h to assemble, to the modern stapler with disposable staple cartridges and simplified hand-controlled, automatic firing mechanisms [1]. The utilization of stapler has been proven to reduce bleeding, postoperative air leak risk, tissue injury, and operative time in many surgery settings. Modern surgical staplers have been widely used for wound closure, organ resection, organ transection, and anastomoses [2]. One of the main applications of a mechanical stapler occurs in pulmonary resection through video-assisted thoracic surgery (VATS), which lacks sufficient surgery space to perform endoscopic suturing [3].

The uneven force distribution inherent in manual stapler operation often causes instability of its distal tip and increases the risk of oozing or bleeding along the staple line. Therefore, a powered stapler was developed to address this limitation by using a motor to power both the staple firing and the cutting action of the blade. A powered stapler can reduce movement at the distal tip by 88% when compared to a manual stapler [4]. In addition, the use of a powered stapler can prevent the operating surgeon’s intraoperative hand tremors thereby minimizing tissue damage. However, there is dissonance among existing literature on the clinical and economic benefits associated with the use of a powered stapler in VATS lobectomy. For example, four real-world studies from the United States, South Korea, and Japan reported that the use of a powered stapler was associated with significantly lower risk of complications (i.e., bleeding and pleurodesis) and lower hospital costs than the use of a manual powered stapler in VATS lobectomy [4–7]. In contrast, three studies from the United States and Europe observed no significant differences in the incidences of complications (e.g., air leak, bleeding, post-surgery adverse events, and length of drainage therapy) between the two types of staplers [8–10]. We recently conducted a retrospective cohort study that observed comparable clinical outcomes associated with the two types of staplers but significantly shorter operation time and length of hospital stay associated with a powered stapler [11]. To further add meaningful evidence to the topic, this study employs the cost-minimization analysis (CMA) method to explore the potential economic benefits associated with using powered stapler in VATS lobectomy for lung cancer.

## Methods

The superior clinical effects of powered staplers over manual staplers in VATS lobectomy for lung cancer have been widely presented [4–7]. To further compare the two types of staplers, this study uses CMA to assess the economic impact of utilizing a powered stapler in VATS lobectomy for lung cancer in a Chinese tertiary hospital. A retrospective cost analysis was conducted to support the development of the CMA model. The ethics approval of this study was obtained from the ethics review board of Xiangya Hospital, which provided de-identified data extracted from the hospital’s medical and billing records associated with patients who underwent VATS lobectomy for lung cancer.

### Retrospective cost analysis

A retrospective cost analysis was conducted to develop predictive models for hospital costs associated with VATS lobectomy for lung cancer in a Chinese tertiary care hospital. The retrospective cost analysis was informed by data from the hospital information system of Xiangya Hospital, a teaching hospital affiliated with Central South University, Changsha, China. The hospital surgical records were screened to identify hospital episodes related to VATS lobectomy for lung cancer from January 1, 2016 to December 31, 2018. This study included patients with routine hospital discharge after VATS lobectomy for lung cancer to control the potential bias that may stem from incomplete information. Patients with extreme values for operation time (> 8 ﻿hours), length of hospital stay (> 21 days), excessive bleeding (> 600 ml), and/or excessive post-surgery drainage volume (> 2400 ml) were excluded to control the potential confounding effects from unusual clinical circumstances. Patients who died during the surgery were excluded as well. Since patients who used imported medical devices for surgery usually had a high socioeconomic status in China [12],  the retrospective cost analysis included only patients who underwent VATS lobectomy using a powered stapler (ECHELON FLEX™ ENDOPATH® Staplers) and a manual stapler (ECHELON FLEX™ Articulating Endoscopic Linear Cutter) imported from the same manufacturer (Ethicon, NJ, USA) to minimize the confounding effects of a patient’s socioeconomic status.

The electronic medical and billing records of the included hospital episodes were used to extract stapler utilization information (e.g., stapler types, numbers of utilized staplers and cartridges), patient baseline characteristics (i.e., demographics, social economic status, lung cancer histology, lung cancer tumor stage, bone marrow function, comorbidity, and operation site), and hospital costs (i.e., total hospital costs and classified hospital costs).

This study divided the included patients into two groups according to the type of stapler used in their surgery (powered stapler group vs. manual stapler group) and compared their baseline characteristics, numbers of staplers and cartridges used during surgery, hospital costs in sub-categories, and total hospital costs. The statistical methods used for these comparisons included a Student’s t-test for continuous data, a Chi-square test for categorical data, and a Wilcoxon rank-sum test for the cost data. To develop predictive models for hospital costs in sub-categories in the CMA, we conducted simple and multiple generalized linear regression using patient characteristics and type of stapler as independent variables and hospital costs, which included disposable supplies costs, drug costs, surgery procedure-related costs, laboratory test costs, and other hospital costs, as dependent variables. Patient characteristics with a significant association with categorized hospital costs in the simple regression analyses were included in the multiple generalized linear regression analyses on the categorized hospital costs.

### Cost-minimization analysis

A decision-analytic model was constructed to simulate hospital costs for VATS lobectomy for lung cancer for two scenarios: utilizing the ECHELON powered stapler and utilizing the ECHELON manual stapler. In each scenario, the model simulated hospital costs associated with a stapler using the predictive models developed from the retrospective cost analysis. The baseline characteristics of the included patients in the retrospective cost analysis were applied to the model cohort in the constructed CMA model. The numbers of staplers and cartridges expended during surgery and their unit prices were used to calculate the acquisition costs of the staplers and cartridges in the two scenarios for the CMA.

The constructed model for CMA was used to conduct the base case analysis, one-way sensitivity analysis, and probabilistic sensitivity analysis (PSA) for the point estimations and uncertainty associated with the differences in simulated hospital costs between the ECHELON powered stapler and the ECHELON manual stapler. The baseline characteristics of the patient cohort from the retrospective cost analysis and the coefficients in the predictive models for the classified hospital costs were applied to run the base case analysis for the point estimations of the differences in total hospital costs and categorized hospital costs between the two scenarios. One-way sensitivity analysis was conducted to assess the impact of uncertainty associated with each model variable on the differences in the estimated total hospital costs associated with the two scenarios. Monte Carlo simulation with 10,000 iterations based on the distributions of coefficients in the cost predictive equations in the CMA model was run to plot the distribution of the differences in the estimated total hospital costs associated with the two scenarios. To demonstrate the differences in total hospital costs between the ECHELON powered stapler and another approved manual stapler brand, this study applied the constructed model for CMA to conduct a scenario analysis comparing the ECHELON powered stapler against the Victor Medical manual stapler—another domestic surgical stapling device approved in Mainland China. Because the ECHELON and the Victor Medical manual staplers shared the same mechanics of operation, the scenario analysis assumed that the two manual staplers had the same hospital costs except for their acquisition costs, which were determined by their unit prices. Accordingly, the scenario analysis used the same predictive equations for the Victor Medical stapler and cartridge on categorized hospital costs in the CMA model to compare the simulated total hospital costs between the ECHELON powered stapler and the Victor Medical manual stapler.

This study used the statistical software R to conduct a retrospective cost analysis. The statistical significance used in the retrospective cost analysis was a two-sided *p*-value less than 0.05. The CMA model was constructed in Microsoft Excel to run the base case analysis, one-way sensitivity analysis, and PSA.

## Results

**Fig. 1:**
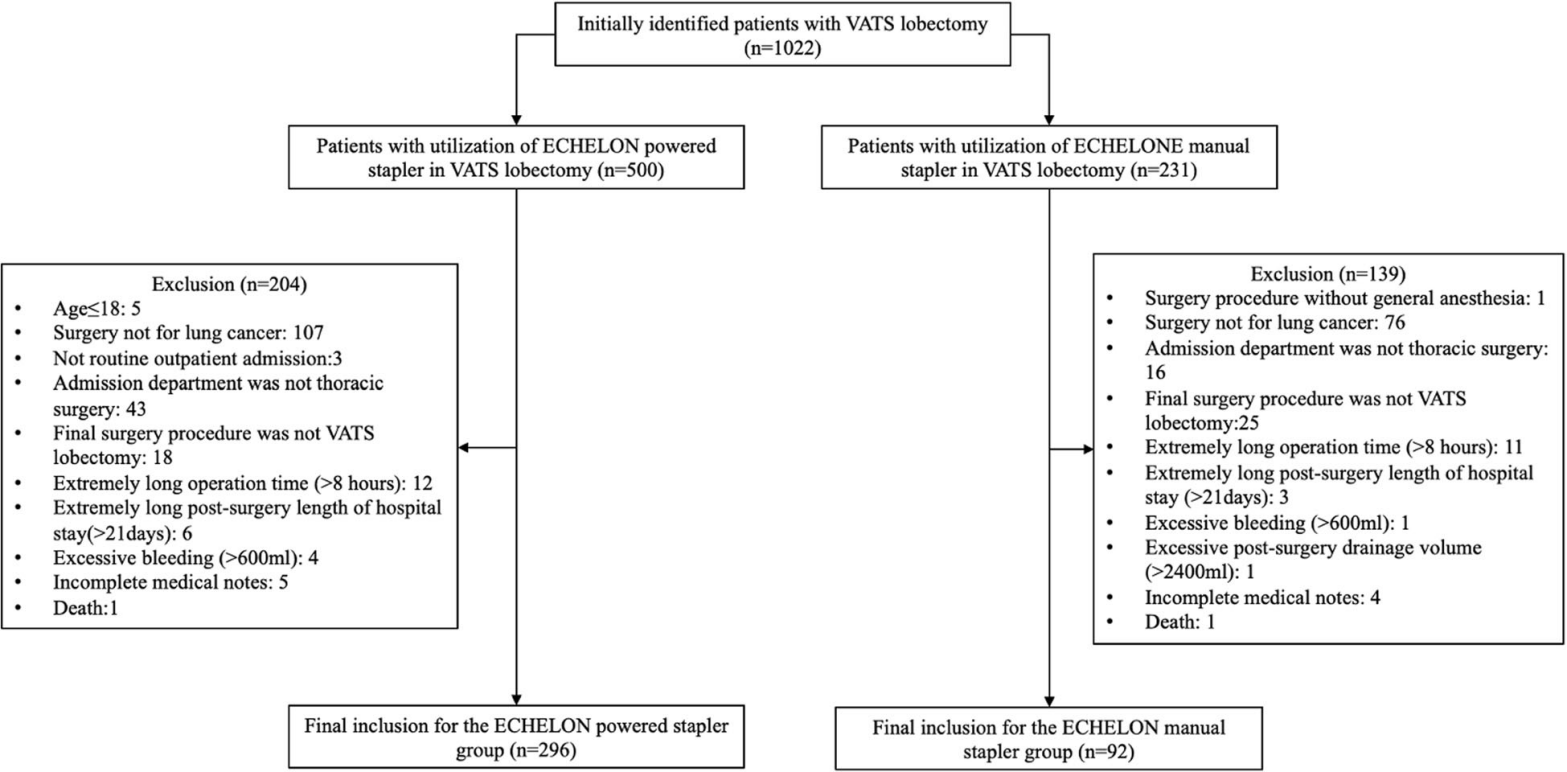
The flow chart of creating two study groups for the retrospective cost analysis comparing ECHELON powered stapler vs. ECHELON manual stapler

The initial search of the hospital surgery records identified 1022 patients who underwent VATS lobectomy for lung cancer during the defined study observation period. Based on the information on the utilization of staplers in VATS lobectomy, this study included 296 patients using the ECHELON powered stapler and 92 patients using the ECHELON manual stapler to conduct the retrospective cost analysis. The patient identification flowchart is illustrated in Fig. 1. The results of the retrospective cost analysis and the CMA comparing the use of a powered stapler with the use of a manual stapler for total hospital costs and categorized hospital costs are summarized in the subsequent sections.

### Retrospective cost analysis: baseline characteristics of the included patients

The baseline characteristics of the included patients were summarized and compared between the two groups: patients whose procedure used the ECHELON powered stapler and those whose procedure used the ECHELON manual stapler. The two study groups had comparable baseline characteristics including age, gender distribution, BMI, and tumor stage distribution. The baseline characteristics with significant differences included area of residence (provincial capital city: 25.4% vs. 14.3%, *p* = 0.025; county: 7.5% vs. 19.8%, *p* = 0.001); urban resident insurance plan (22.0% vs. 7.7%, *p* = 0.019); abnormal erythrocyte counting (24.4% vs. 14.3%, *p* = 0.043); lobectomy site at bottom left lung (11.5% vs. 24.2%, *p* = 0.016); and the comorbidities that included bronchial disease (13.5% vs. 3.3%, *p* = 0.006), coronary heart disease (2.7% vs. 9.8%, *p* = 0.004), and sport system diseases (9.8% vs. 3.3%, *p* = 0.047). The baseline patient characteristics of the two study groups are summarized in Table 1.

**Table 1:** Summary of patient characteristics of the created two stapler groups in the retrospective cost analysis

Patient characteristics	ECHELON powered stapler group (*N* = 296)	ECHELON manual stapler group (*N =* 92)	*P* value
	%/Mean+/−SD	%/Mean+/−SD	
Demographics			
Age (years)	57.9+/−8.9	58.5+/−9.0	0.823
Male gender	53.0%	63.0%	0.092
BMI (kg/m^2^)	23.8+/−3.1	23.5+/−2.8	0.343
BMI < 18.5	3.8%	0.0%	0.609
18.5 < =BMI < 24	50.2%	62.4%	0.168
24 < =BMI < 28	36.9%	27.5%	0.280
28 < =BMI < 30	6.4%	7.6%	0.730
30 < =BMI < 40	2.7%	2.5%	1.000
Place of residence			
Provincial capital city	25.4%	14.3%	***0.025***
Prefecture-level city	59.3%	60.4%	0.910
County	7.5%	19.8%	***0.001***
Other cities	7.8%	5.5%	0.450
Public insurance plan			
Urban employee medical insurance	27.1%	19.2%	0.294
Urban resident medical insurance	22.0%	7.7%	***0.019***
New rural cooperative medical insurance	27.1%	36.5%	0.180
Others	23.7%	36.5%	0.080
Marital status			
Married	99.3%	100.0%	1.000
Single	0.7%	0.0%	1.000
Comorbidity			
Digestive system diseases	43.9%	38.0%	0.319
Urinary system diseases	23.0%	16.3%	0.173
Hypertension	20.3%	17.4%	0.543
Reproductive system diseases	17.2%	13.0%	0.342
Cerebrovascular/cerebrovascular diseases	15.9%	17.4%	0.731
Bronchial diseases	13.5%	3.3%	***0.006***
Endocrine system diseases	11.1%	7.6%	0.329
Sports system diseases	9.8%	3.3%	***0.047***
Diabetes	9.1%	8.7%	0.901
Heart disease	8.8%	12.0%	0.365
Emphysema	7.4%	7.6%	0.955
Bullae	5.1%	5.4%	0.889
Coronary heart disease	2.7%	9.8%	***0.004***
Tumor histology			
Squamous cell carcinoma	14.6%	14.8%	1.000
Adenocarcinoma	78.2%	81.5%	0.809
Other	7.1%	3.7%	0.792
Tumor stage			
Carcinoma in situ	1.0%	0.0%	0.332
I	56.5%	47.8%	0.148
II	11.8%	17.4%	0.167
III	29.7%	33.7%	0.471
IV	1.0%	1.1%	0.951
Bone marrow function			
Abnormal INR	3.9%	3.5%	1.000
Abnormal hemoglobin	15.9%	24.2%	0.085
Abnormal erythrocyte counts	24.4%	14.3%	***0.043***
Abnormal leukocyte counts	8.5%	9.9%	0.675
Abnormal platelet counts	7.5%	12.1%	0.197
Lobectomy site			
Upper right	38.1%	25.8%	0.082
Right middle	7.5%	3.0%	0.268
lower right	14.3%	24.2%	0.062
Upper middle right	0.8%	0.0%	1.000
Lower middle right	1.6%	1.5%	1.000
Left lung	0.4%	0.0%	1.000
Upper left	25.8%	21.2%	0.523
Bottom left	11.5%	24.2%	***0.016***

### Retrospective cost analysis: number of used staplers and cartridges and categorized hospital costs

The number of staplers used during surgery in the ECHELON powered stapler group and the ECHELON manual stapler group were highly comparable (1.2+/− 0.6 vs. 1.2+/− 0.5, *p* = 0.627). However, the powered stapler group used fewer cartridges than the manual stapler group (7.0+/− 3.9 vs. 7.5+/− 4.0, *p* = 0.076). Compared with the ECHELON manual stapler group, the ECHELON powered stapler group had lower hospital costs for drugs (median: ¥10,161 vs. ¥13,592, *p* < 0.001) but higher operation-related hospital costs (median: ¥8257 vs. ¥7683, p < 0.001). The total hospital costs of the ECHELON powered stapler group were significantly lower than those incurred by the ECHELON manual stapler group (median: ¥64,322 vs. ¥67,298, p < 0.001) because the drug costs saved from the use of the powered stapler were higher than the sum of the powered stapler group’s increased operation-related hospital costs and stapler acquisition costs. The number of staplers used during surgery and categorized hospital costs for the two study groups are summarized in Table 2.

**Table 2:** Summary of the utilizations of staplers and classified hospital costs associated with VATS lobectomy for lung cancer in the created two stapler groups

Study group	ECHELON powered stapler				ECHELON manual stapler				P value
Sample size	296				92				
Outcomes	Mean	SD	Median	Q1-Q3	Mean	SD	Median	Q1-Q3	
Utilized number of staplers and cartridges									
Staplers	1.2	0.6			1.2	0.5			0.627
Cartridges	7.0	3.9			7.5	4.0			0.076
Classified hospital costs									
Staplers costs	¥24,645	¥10,592	¥21,437	(¥18,996–¥26,319)	¥23,255	¥10,291	¥21,058	(¥16,845–¥24,570)	0.656
Disposable supplies costs	¥9853	¥3022	¥9913	(¥7769–¥11,793)	¥10,518	¥3638	¥10,358	(¥8707–¥12,064)	0.080
Drug costs	¥10,079	¥4504	¥10,161	(¥7737–¥9212)	¥14,066	¥5352	¥13,592	(¥7298–¥8395)	***< 0.001***
Operation related costs	¥7743	¥2279	¥8257	(¥1780–¥2731)	¥7523	¥1731	¥7683	(¥1834–¥2576)	***< 0.001***
Laboratory tests costs	¥10,264	¥4209	¥10,323	(¥59,307–¥70,450)	¥11,215	¥3723	¥10,900	(¥63,141–¥74,624)	0.077
Other utilized hospital resources costs	¥2253	¥892	¥2269	(¥1780–¥2731)	¥2229	¥743	¥2179	(¥1834–¥2576)	0.524
Total hospital costs	¥64,836	¥8928	¥64,322	(¥59,307–¥70,450)	¥68,806	¥10,616	¥67,298	(¥63,141–¥74,624)	***< 0.001***

### Retrospective cost analysis: developing predictive equations for categorized hospital costs

Our multiple generalized regression analysis identified that the categorized hospital costs for disposable supplies were significantly associated with BMI distribution (18.5 < =BMI < 24: coefficient − 0.099, *p* = 0.007; 24 < =BMI < 28: coefficient − 0.133, *p* = 0.001; 30 < =BMI < 40: coefficient − 0.388, *p* = 0.002) and the following comorbidities: lung infection (coefficient − 0.286, *p* = 0.004), tuberculosis (coefficient − 0.205, *p* = 0.027), breast diseases (coefficient − 0.524, *p* = 0.015). The categorized hospital costs for drugs were significantly associated with the use of a powered stapler (coefficient − 0.256, *p* < 0.001), tumor stage I (coefficient − 0.153, p < 0.001), BMI distribution for patients with 24 < =BMI < 28 (coefficient − 0.148, *p* = 0.005) and patients with 30 < =BMI < 40 (coefficient − 0.330, *p* = 0.002), and the following comorbidities: urological diseases (coefficient 0.144, p = 0.005), endocrine diseases (coefficient − 0.164, *p* = 0.019), and abnormal platelet counts (coefficient 0.233, p = 0.002). Additionally, the categorized hospital costs for operation were significantly associated with the following comorbidities: bronchial diseases (coefficient 0.108, *p* = 0.025), urological diseases (coefficient 0.074, *p* = 0.045), and immune system diseases (coefficient − 0.393, *p* = 0.019). The categorized hospital costs for laboratory tests were significantly associated with BMI distribution for patients with 18.5 < =BMI < 24 (coefficient 0.100, *p* = 0.024), unspecified insurance plan (coefficient: 0.124, *p =* 0.019), and the following comorbidities: diabetes (coefficient: 0.165, *p =* 0.019), urological diseases (coefficient 0.133, *p* = 0.007), and immune system diseases (coefficient − 0.521, *p* = 0.023). Lastly, the categorized hospital costs for other utilized resources were significantly associated with tumor stage I (coefficient − 0.083, *p* = 0.033) and the following comorbidities: diabetes (coefficient 0.158, *p* = 0.020) and urological diseases (coefficient 0.176, *p* < 0.001). The results of the multiple generalized regression analyses for the categorized hospital costs are summarized in Table 3*.*

**Table 3:** The results of the multiple generalized linear regression analyses exploring the predictors for the classified hospital costs associated with VATS lobectomy for lung cancer in a Chinese tertiary care hospital

A Hospital costs for disposable supplies				
Hospital cost classification	Hospital costs for disposable supplies			
Variables	Coefficient	95% CI		P value
		Lower	Upper	
Intercept	9.279	9.203	9.357	***< 0.001***
Powered stapler vs. Manual stapler	−0.007	− 0.084	0.069	0.854
Demographics				
Male vs. female	0.041	−0.048	0.129	0.361
BMI				
18.5 < =BMI < 24 vs. Other BMI distribution ranges	−0.099	− 0.171	− 0.028	***0.007***
24 < =BMI < 28 vs. Other BMI distribution ranges	−0.133	− 0.214	− 0.052	***0.001***
30 < =BMI < 40 vs. Other BMI distribution ranges	−0.388	− 0.624	− 0.134	***0.002***
Tumor histology				
Adenocarcinoma vs. Non-adenocarcinoma	−0.041	− 0.108	0.025	0.225
Tumor stage				
Stage I vs. Non-stage I	0.058	−0.005	0.120	0.072
Bone marrow function				
Abnormal INR vs. Normal INR	0.141	−0.022	0.311	0.099
Abnormal hemoglobin vs. Normal hemoglobin	0.076	− 0.004	0.157	0.066
Lobectomy site				
Upper left vs. Non-upper left site	−0.026	− 0.100	0.050	0.500
Comorbidity				
Hypertension vs. Non-hypertension	−0.051	− 0.128	0.026	0.193
Immune system diseases vs. Non-immune system diseases	0.177	−0.193	0.583	0.355
Breast diseases vs. Non-breast diseases	−0.524	− 0.917	− 0.075	***0.015***
B. Hospital for drugs				
Hospital cost classification	Hospital costs for drugs			
Variables	Coefficient	95% CI		P value
		Lower	Upper	
Intercept	9.492	9.337	9.649	***< 0.001***
Powered stapler vs. Manual stapler	−0.256	−0.375	−0.139	***< 0.001***
Demographics				
Male vs. female	0.041	−0.048	0.129	0.361
BMI				
24 < =BMI < 28 vs. Other BMI distribution ranges	−0.148	− 0.250	− 0.044	***0.005***
28 < =BMI < 30 vs. Other BMI distribution ranges	−0.330	− 0.535	− 0.112	***0.002***
Residence city				
County vs. Other cities	0.134	−0.002	0.273	0.058
Marital status				
Married vs. Other marital status	−0.010	−0.129	0.106	0.864
Public health insurance plan				
Urban worker insurance plan vs. other insurance plan	−0.049	− 0.155	0.058	0.367
Tumor histology				
Adenocarcinoma vs. Non-adenocarcinoma	0.018	−0.074	0.109	0.702
Tumor stage				
Stage I vs. Non-stage I	−0.153	−0.240	− 0.067	***< 0.001***
Bone marrow function				
Abnormal platelet counts vs. normal platelet counts	0.233	0.085	0.386	***0.002***
Lobectomy site				
Upper left vs. Non-upper left site	0.029	−0.071	0.131	0.576
Comorbidity				
Diabetes vs. Non-diabetes	0.124	−0.017	0.270	0.091
Cerebrovascular diseases vs. Non-cerebrovascular diseases	0.095	−0.018	0.210	0.103
Urological diseases vs. Non-urological diseases	0.144	0.045	0.246	***0.005***
Cardiovascular diseases vs. Non-cardiovascular diseases	0.073	−0.066	0.217	0.310
Endocrine diseases vs. Non-endocrine diseases	−0.164	− 0.297	− 0.026	***0.019***
C. Hospital costs related to operation				
Hospital cost classification	Hospital costs related to operation			
Variables	Coefficient	95% CI		P value
		Lower	Upper	
Intercept	8.925	8.856	8.994	***< 0.001***
Powered stapler vs. Manual stapler	0.004	−0.065	0.072	0.909
Tumor stage				
Stage I vs. Non-stage I	−0.052	− 0.110	0.006	0.079
Lobectomy site				
Upper left vs. Non-upper left site	0.002	−0.070	0.075	0.954
Comorbidity				
Bronchial diseases vs. Non-bronchial diseases	0.108	0.015	0.203	***0.025***
Immune system diseases vs. Non-immune system diseases	−0.393	− 0.707	− 0.047	***0.019***
Cerebrovascular diseases vs. Non-cerebrovascular diseases	0.040	−0.038	0.120	0.316
Urological diseases vs. Non-urological diseases	0.074	0.002	0.146	***0.045***
Cardiovascular diseases vs. Non-cardiovascular diseases	0.066	−0.032	0.167	0.196
Sport system diseases vs. Non-sport system diseases	0.071	−0.033	0.178	0.186
Vascular diseases vs. Non-vascular diseases	0.088	− 0.142	0.334	0.472
D. Hospital costs for laboratory tests				
Hospital cost classification	Hospital costs for laboratory tests			
Variables	Coefficient	95% CI		P value
		Lower	Upper	
Intercept	9.133	8.851	9.417	***< 0.001***
Powered stapler vs. Manual stapler	−0.094	−0.203	0.014	0.082
Demographics				
Age	0.002	−0.003	0.007	0.367
18.5 < =BMI < 24 vs. Other BMI distribution ranges	0.100	0.013	0.188	***0.024***
Public health insurance plan				
Urban worker insurance plan vs. Other insurance plans				
New rural cooperative medical insurance vs. Other insurance plans	−0.029	− 0.128	0.071	0.562
Tumor histology				
Adenocarcinoma vs. Non-adenocarcinoma	−0.031	− 0.117	0.055	0.480
Tumor stage				
Stage I vs. Non-stage I	−0.033	− 0.113	0.048	0.427
Bone marrow function				
Abnormal white cell counts vs. Normal white cell counts	−0.117	−0.256	0.026	0.101
Lobectomy site				
Upper left vs. Non-upper left site	0.014	−0.082	0.111	0.781
Comorbidity				
Immune system diseases vs. Non-immune system diseases	−0.521	− 0.944	− 0.039	***0.023***
Diabetes vs. Non-diabetes	0.165	0.030	0.305	***0.019***
Cerebrovascular diseases vs. Non-cerebrovascular diseases	0.046	−0.063	0.157	0.415
Urological diseases vs. Non-urological diseases	0.133	0.038	0.230	***0.007***
Cardiovascular diseases vs. Non-cardiovascular diseases	0.088	−0.046	0.225	0.206
E. Other hospital costs				
Hospital cost classification	Other hospital costs			
Variables	Coefficient	95% CI		P value
		Lower	Upper	
Intercept	7.707	7.618	7.798	***< 0.001***
Powered stapler vs. Manual stapler	−0.008	−0.099	0.080	0.854
Tumor stage				
Stage I vs. Non-stage I	−0.083	−0.159	− 0.007	***0.033***
Lobectomy site				
Upper left vs. Non-upper left	−0.019	−0.111	0.075	0.684
Comorbidity				
Immune system diseases vs. Non-immune system diseases	−0.410	−0.817	0.054	0.064
Diabetes vs. Non-diabetes	0.158	0.028	0.292	***0.020***
Cerebrovascular diseases vs. Non-cerebrovascular diseases	0.068	−0.033	0.172	0.195
Urological diseases vs. Non-urological diseases	0.176	0.084	0.269	***< 0.001***

### Cost-minimization analysis: base case analysis

The following values were used to conduct the base case analysis: (1) baseline characteristics of the included patients at an individual level in the retrospective cost analysis; (2) the unit prices of the ECHELON powered stapler (PSE45A stapler: ¥6790, ECR45B cartridge: ¥2441) and the ECHELON manual stapler (EC45A stapler: ¥3970, ECR45B cartridge: ¥2441); and (3) the baseline coefficients of the predictive equations for the categorized hospital costs. As a result, the base case CMA estimated that the total hospital costs associated with the utilization of the ECHELON powered stapler and the ECHELON manual stapler were ¥65,531 and ¥67,184, respectively. The comparison of the distributions of hospital costs associated with the two types of staplers in the CMA suggested that the powered stapler saved the total hospital costs mainly through reducing costs incurred for cartridges, laboratory tests, and drugs. The results of the base case CMA are summarized in Table 4.

**Table 4:** Summary of the results of base case CMA comparing ECHELON powered stapler vs. two manual staplers for hospital costs associated with VATS lobectomy for lung cancer in a Chinese tertiary care hospital

Cost classification	ECHELON powered stapler	ECHELON manual stapler	Victor Medical manual stapler	Difference (ECHELON powered stapler vs. ECHELON manual stapler)	Difference (ECHELON powered stapler vs. Victor Medical manual stapler)
Stapler	¥8327	¥4790	¥4882	¥3537	¥3445
Cartridge	¥17,129	¥18,308	¥20,974	-¥1179	-¥3845
Disposable supplies	¥9918	¥9989	¥9989	-¥72	-¥72
Drugs	¥10,095	¥13,043	¥13,043	-¥2948	-¥2948
Operation related	¥7674	¥7643	¥7643	¥31	¥31
Laboratory tests	¥10,159	¥11,162	¥11,162	-¥1003	-¥1003
Others	¥2229	¥2248	¥2248	-¥19	-¥19
Total hospital costs	¥65,531	¥67,184	¥69,942	-¥1653	-¥4411

### Cost-minimization analysis: sensitivity analyses

**Fig. 2:**
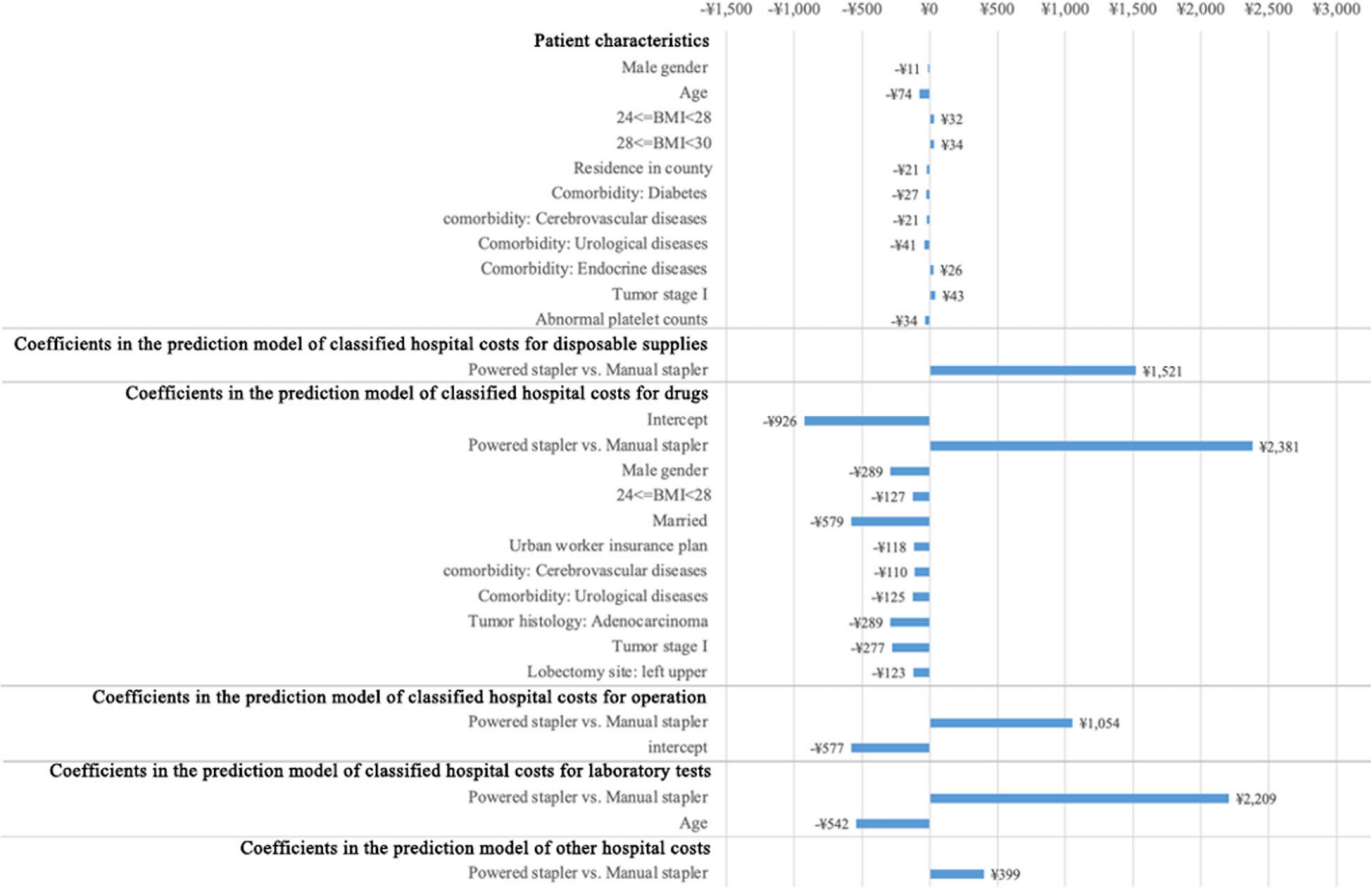
The results of one-way sensitivity analyses comparing the hospital costs associated with powered and manual stapler in VATS lobectomy for lung cancer

The one-way sensitivity analyses found that the uncertainty associated with patient characteristics had a limited impact on the differences in total hospital costs between the powered stapler model scenario and the manual stapler model scenario in CMA. The coefficients associated with the stapler type in the predictive models were the driving variables that could increase the difference in total hospital costs between the two groups from ¥309 to ¥2381. The results of the one-way sensitivity analyses are illustrated in Fig. 2.

**Fig. 3:**
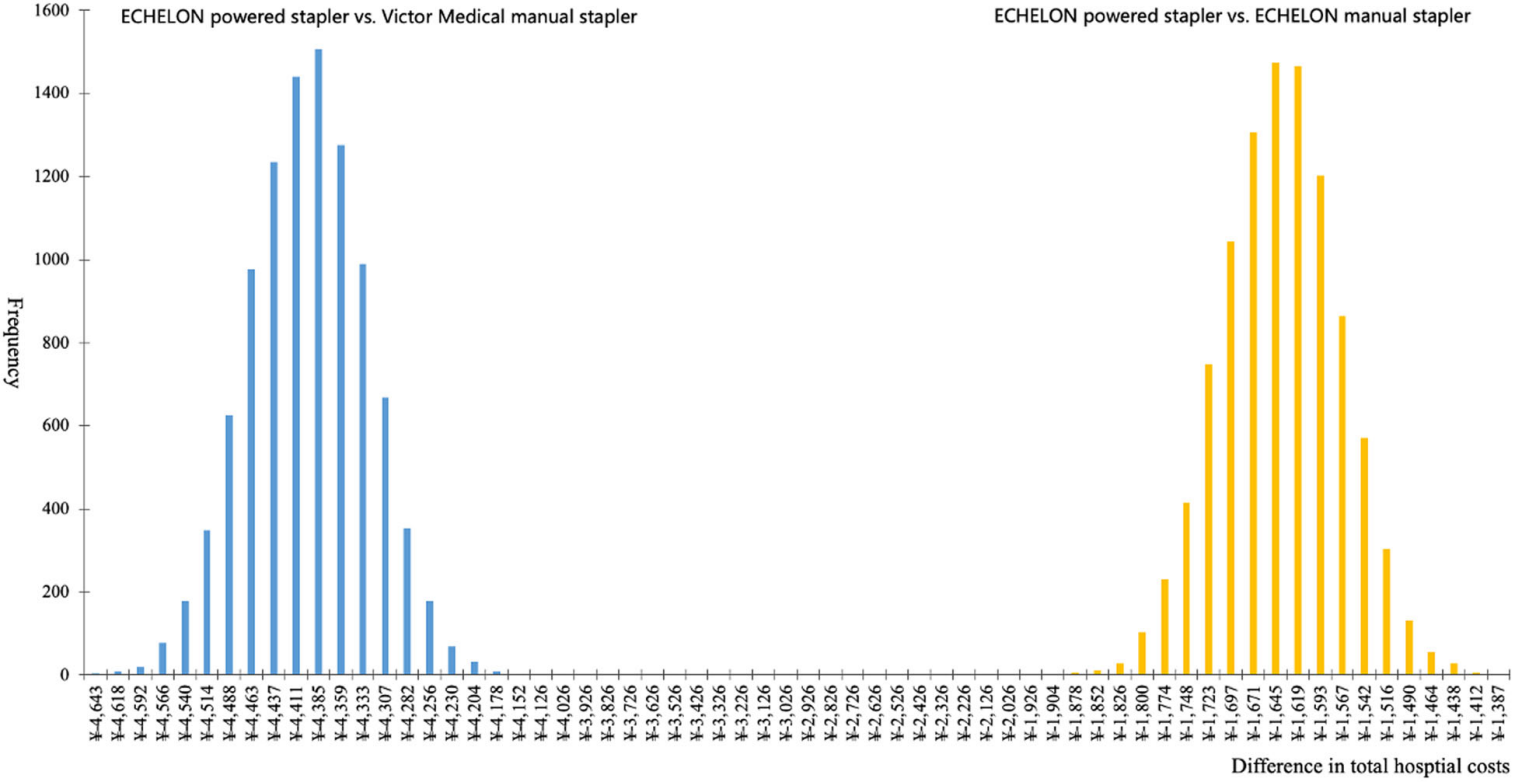
The distribution of the 10,000 Monte Carlo simulations for the differences in total hospital costs associated with ECHELON powered stapler and the two manual staplers in VATS lobectomy for lung cancer in a Chinese tertiary care hospital

Based on the outputs of 10,000 Monte Carlo simulations in the CMA model, the median difference in the estimated total hospital costs between the ECHELON powered stapler and the ECHELON manual stapler was -¥1651 (95% credible interval: -¥1787 to -¥1520). The ECHELON powered stapler presented a 100% probability to save total hospital costs when considering the current unit prices of the staplers and cartridges and the overall uncertainty associated with patient characteristics and coefficients in the predictive equations developed for categorized hospital costs. The distribution of the simulated differences in total hospital costs of the two staplers are plotted in Fig. 3.

### Cost-minimization analysis: scenario analyses

The ECHELON manual stapler was replaced with the Victor Medical manual stapler in the CMA, which had a cheaper unit price (¥3800) but a more expensive cartridge (¥2987), to conduct the scenario analysis. The scenario analysis indicated that the ECHELON manual stapler was associated with lower point estimation for total hospital costs than the Victor Medical manual stapler (¥65,531 vs. ¥69,942). The scenario analysis used the same predictive equations for categorized hospital costs; therefore, the replacement of the ECHELON manual stapler with the Victor Medical manual stapler had no influence on the impact of uncertainty associated with the variables of the CMA model in the one-way sensitivity analysis. The 10,000 Monte Carlo simulations using the new CMA model were plotted to identify the median difference in total hospital costs between the ECHELON powered stapler and the Victor Medical manual stapler (− ¥4409, 95% credible interval: -¥4545 to -¥4278).

## Discussion

The economic advantages of powered stapler use in VATS lobectomy for lung cancer have been previously reported in retrospective studies from the United States [5] and Korea [6]. Like previous cost studies, our retrospective cost analysis presents significantly lower total hospital costs associated with the use of a powered stapler in China. Therefore, the findings of our study reaffirm the existing pool of economic evidence and external validity for powered stapler use in VATS lobectomy for lung cancer. In addition, a cost-minimization model was implemented in this study to assess the robustness of the findings under the overall uncertainty of model variables. Consequently, the findings of this study can be assuredly used to support hospital budget planning and reimbursement decision making.

The majority of the patients included in this study (76.3%) underwent VATS lobectomy for lung cancer using a powered stapler. Although this study did not survey surgeons for their preferences for the stapler type, the observed differences in patient characteristics in the two study groups suggest the powered stapler as the preferred option for surgeons due to its operational advantages. For example, the greater operability of a powered stapler than a manual stapler in VATS with limited surgical space would lead surgeons to use a powered stapler on patients with a high BMI who often presents further spatial constraints [13, 14]. In addition, the even force distribution of a powered stapler could reduce lung damage [15] and allow surgeons to operate on complex anatomical sites that require precise and delicate operation [16]. Our retrospective cost analysis observed significantly lower total hospital costs associated with the use of a powered stapler as corroborated by previous studies [5, 6]. Both unadjusted and adjusted comparisons of the classified hospital costs of the two stapler types identified that the powered stapler saved sufficient hospital drug costs to fully offset the increase in costs for the acquisition costs of a powered stapler and matching cartridges. This study did not further investigate the utilization of drugs in the two stapler groups. However, our previous study with the same patient cohort found that the powered stapler was associated with significantly shorter post-surgery hospital length of stay [11], which could partially explain the lower hospital drug costs observed in the powered stapler group.

This study additionally assessed the robustness of its findings under the overall uncertainty of patient characteristics using CMA. The one-way sensitivity analysis of CMA found that patient characteristics had a minimal impact on the differences in the total hospital costs of the two stapler groups. These differences were mainly driven by the acquisition costs of the staplers and matching cartridges. The PSA, which is used to translate model parameter uncertainty into outcome values and decision uncertainty, reported 100% probability for the powered stapler to save total hospital costs in the model cohort. This, therefore, verifies the robustness of our finding that lower total hospital costs were associated with powered stapler use in VATS lobectomy for lung cancer. In comparison to the retrospective cost analysis—the most common method used in cost studies—the CMA employed in our study has several advantages. First, the simulation was conducted on the entire patient cohort for each stapler type, resulting in a substantial improvement in the model cohort size and reduction in the uncertainty of model outputs for cost outcomes. Next, the use of a paired test in the comparison of the simulated cost outcomes associated with the two staplers in the model cohort excluded any confounding effects in the analysis. Another advantage is that our CMA allowed for the investigation of the impact of the uncertainty of model variables on model outputs through one-way sensitivity analysis and PSA. Our study findings that have gone through the uncertainty assessment can thus better inform the decision making in both clinical and reimbursement contexts. Lastly, the CMA can be adapted to conduct scenario analyses using other types of staplers. For example, we applied the constructed model for CMA to conduct a scenario analysis comparing the powered stapler with another type of manual stapler. After adjusting the prices of the stapler and cartridge, we were able to easily estimate the differences in total hospital costs between the powered stapler and another type of manual stapler.

Our study has several implications on clinical practice, research methods, and policy making. To our knowledge, the economic impact of powered stapler use in VATS lobectomy has never been assessed as comprehensively as in the present study. The identified differences in hospital costs associated with the two stapler types could inform the selection of a stapler type for clinical procedures using an economic lens, which is crucial for reimbursement decision making, hospital budget control, and patient affordability in China. Additionally, our study demonstrates that health economic evaluations should focus on the total costs associated with the complete pathway of the management but not the acquisition costs of the interventions. New technologies are often more costly. However, the potential clinical benefits of such new technologies could have positive economic implications that offset their high costs. In our case, the ECHELON powered stapler cost more than the manual stapler to purchase but incurred lower overall hospital costs. Furthermore, our study suggests that medical costs could be an alternative outcome measure to address the measurement bias frequent in retrospective studies. The lack of standard data collection method for medical information could substantially confound the comparisons of interventions in real-world settings. With growing utilization of real-world evidence in reimbursement decision making and health policy development, measurement bias associated with real-world evidence from retrospective studies should be carefully assessed and economic outcomes should be always considered for validation in real-world studies. Finally, our study demonstrates a great use case of CMA in informing health policy making. The constructed model for CMA can be adapted as a policy making tool to guide policymakers in understanding the cost rationales and the uncertainty of the economic benefits of powered stapler use. CMA can be applied to studying a wide array of stapler types and settings with appropriate adjustments.

Common limitations inherent in the retrospective research method were found in this present study. Despite the low probability of missing data on medical costs and information on stapler utilization as a result of using hospital billing records, there is chance that patients’ medical records may be missing information that could confound the cost comparison analyses between the two stapler groups. For example, the medical records did not collect sufficient information to adjust for residual confounding in our data analyses because powered stapler use was likely more attractive to surgeons for use in VATS lobectomy and patients with higher socioeconomic status may prefer to receive more expensive and advanced medical devices [17]. Therefore, a prospective study design could be more appropriate than a retrospective design to study the economic benefits of a powered stapler. Additionally, the assumption that the ECHELON manual stapler and the Victor Medical manual stapler share the same clinical effects and health resource utilization in the scenario analysis should be confirmed in the future. Interpreting evidence from the scenario analysis therefore requires caution.

## Conclusions

This study conducted a retrospective cost analysis to develop a CMA model to demonstrate the economic impact of the ECHELON powered stapler in comparison to the ECHELON manual stapler in VATS lobectomy conducted for early-stage lung cancer in a Chinese tertiary hospital. With a full adjustment for the patient characteristics from historical medical records, the use of the ECHELON powered stapler could cost less than two approved manual staplers in China as evinced by a base case analysis and sensitivity analyses. Utilizing a powered stapler in VATS lobectomy for lung cancer could support health budget controls in Chinese tertiary hospitals.

## Supplementary Information

**Additional file 1: Supplemental Table**. Summary of the prediction formulas for categorized hospital costs in the cost-minimization model

## Data Availability

The data can be obtained through the permission from data owner, Xiangha Hospital.

## Abbreviations

CMA: Cost-minimization analysis
PSA: Probabilistic sensitivity analysis
VATS: Video-assisted thoracic surgery

## References

[1] Akopov A, Artioukh DY, Molnar TF (2021). Surgical staplers: the history of conception and adoption. Ann Thorac Surg.

[2] Akopov AL, Artioukh DY, Molnar TF (2020). History of mechanical staple surgical suture (review of literature). Grekov's Bulletin of Surgery.

[3] Berfield KS, Farjah F, Mulligan MS (2019). Video-assisted thoracoscopic lobectomy for lung cancer. Ann Thorac Surg.

[4] Tsunezuka Y, Tanaka N, Fujimori H (2020). The impact of endoscopic stapler selection on bleeding at the vascular stump in pulmonary artery transection. Medical Devices (Auckland, NZ).

[5] Miller DL, Roy S, Kassis ES, Yadalam S, Ramisetti S, Johnston SS (2018). Impact of powered and tissue-specific endoscopic stapling technology on clinical and economic outcomes of video-assisted thoracic surgery lobectomy procedures: a retrospective, observational study. Adv Ther.

[6] Park SY, Kim DJ, Mo Nam C, Park G, Byun G, Park H, Choi JH (2019). Clinical and economic benefits associated with the use of powered and tissue-specific endoscopic staplers among the patients undergoing thoracoscopic lobectomy for lung cancer. J Med Econ.

[7] Shigeeda W, Deguchi H, Tomoyasu M, Kudo S, Kaneko Y, Kanno H, Saito H (2021). Utility of the powered stapler for radical pulmonary resection: a propensity score-matched analysis. Surg Today.

[8] Licht PB, Ribaric G, Crabtree T, Lanuti M, Molins L, Knippenberg S, Schwiers M, Yoo A (2015). Prospective clinical study to evaluate clinical performance of a powered surgical stapler in video-assisted thoracoscopic lung resections. Surg Technol Int.

[9] Molins L, Lanuti M, Force S, Woolley S, Krantz S, Creedon EE, Schwiers ML, Singleton DW, Waggoner JR, Fryrear R, Licht P (2020). Evaluation of a powered vascular stapler in video-assisted thoracic surgery lobectomy. J Surg Res.

[10] Akil A, Semik M, Freermann S, Reichelt J, Redwan B, Görlich D, Fischer S (2019). Use of a powered stapling system for minimally invasive lung volume reduction surgery: results of a prospective double-blind single-center randomized trial. Thorac Cardiovasc Surg.

[11] Gao Y, Xiong F, Xia X, Gu P, Wang Q, Wu A, Zhan H, Chen W, Qian Z (2021). Clinical outcomes of powered and manual staplers in video-assisted thoracic surgery lobectomy for lung cancer. J Comp Effectiveness Res.

[12] Liu GG, Zhao Z, Cai R, Yamada T, Yamada T (2002). Equity in health care access to: assessing the urban health insurance reform in China. Soc Sci Med.

[13] St Julien JB, Aldrich MC, Sheng S, Deppen SA, Burfeind WR, Putnam JB (2012). Obesity increases operating room time for lobectomy in the society of thoracic surgeons database. Ann Thorac Surg.

[14] Ji Y, Qiu B, Gao S (2019). The powered vascular staple (PVS) versus conventional powered linier cutter (PLC) for the application of bronchial transection in thoracoscopic anatomic segmentectomy. J Thorac Dis.

[15] Ng CS, Pickens A, Siegel JM, Clymer JW, Cummings JF (2016). A novel narrow profile articulating powered vascular stapler provides superior access and haemostasis equivalent to conventional devices. Eur J Cardiothorac Surg.

[16] Kang GJ, Jiang WY, Xie SP, Huang J (2013). VATS right upper lobectomy. J Thorac Dis.

[17] Gong Y, Yin X, Wang Y, Li Y, Qin G, Liu L, Zhou W, Song F, Dong X, Cao S, Yang C, Yang H, Xie J, Liu J, Lu Z (2014). Social determinants of community health services utilization among the users in China: a 4-year cross-sectional study. PLoS One.

