# Supplementary material for: Economic impact of powered stapler in video-assisted thoracic surgery lobectomy for lung Cancer in a Chinese tertiary hospital: a cost-minimization analysis
Source: Health Econ Rev. 2022 Feb 9;12:12. doi: 10.1186/s13561-022-00359-x (PMC8830080; doi:10.1186/s13561-022-00359-x)
Supplement: Supplementary file 1 — Additional file 1: Supplemental Table. Summary of the prediction formulas for categorized hospital costs in the cost-minimization model [file 13561_2022_359_MOESM1_ESM.docx]

Supplemental table: Summary of the prediction formulas for categorized hospital costs in the cost-minimization model.

| Categorized hospital costs | Prediction formula |
| --- | --- |
| Disposable supplies | exp(9.279-0.007*stapler type-0.099*BMI category for 18.5 to 24-0.133*BMI category for 24 to 28-0.388*BMI category for 30 to 40-0.286*lung infection-0.205*lung tubeclerous-0.051*hypertension+0.177*immune system diseases-0.524*breast diseases-0.098*other comorbidities-0.041*adenocarcinoma histology+0.238*other non-tumor histology+0.058*tumor stage I+0.141*abnormal INR+0.076*abnormal hemoglobin -0.026*operation site at upper left lung) |
| Drugs | exp(9.492-0.256*stapler type+0.041*male gender-0.148*BMI category for 24to 28)-0.330*BMI category for 28 to 30-0.010*married status+0.134*residence in county-0.049*urban insurance plan+0.124*diabetes+0.095*cerebrovascular diseases+0.144*urological diseases+0.073*heart diseases-0.164*endocrinological diseases+0.018*adenocarcinoma histology-0.153*tumor stage I+0.233*abnormal platelet +0.036*operation classification III+0.029* operation site at upper left lung) |
| Operation | exp(8.925+0.004*stapler type+0.108*bronchial diseases +0.040*cerebrovascular diseases+0.074*urological diseases+0.066*heart diseases+0.071*sport system diseases+0.088*vascular diseases-0.393*immune system diseases-0.052*tumor stage I+0.002*operation site at upper left lung) |
| Laboratory tests | exp(9.133-0.094*stapler type+0.002*age+0.100*BMI category for 18.5 to 24-0.029*rural farmer insurance plan+0.124*other insurance plan+0.165*diabetes+0.046*cerebrovascular+0.133*urological diseases+0.088*heart diseases-0.521*immune system diseases-0.031*adenocarcinoma histology-0.033*tumor stage I-0.117*abnormal white cell counts+0.009*operation classification III+0.014*operation site at upper left lung) |
| Other utilized hospital resources | exp(7.707-0.008*stapler type+0.158*diabetes+0.068*cerebrovascular diseases+0.176*urological diseases-0.410*immune system diseases-0.083*tumor stage I-0.019*operation site at upper left lung) |
